# Comparison of a Smartphone-Based Method for Measuring Anterior Chamber Depth with Anterior Segment OCT and Smith’s Technique

**DOI:** 10.3390/jcm15187048

**Published:** 2026-09-11

**Authors:** Heather R. M. Connor, Luke X. Chong, Kingsley Chung, Brooke Daws, Monica Hanna, Monique Jankovski, Minh Luu, Sarah Mawdsley, Madi Pollock, Cameron Skinner, Jessie Whiley, Jessica Xu, Amanda K. Edgar

**Affiliations:** 1 School of Medicine (Optometry), Faculty of Health, Deakin University, 75 Pigdons Road, Waurn Ponds 3216, Australia; luke.chong@deakin.edu.au (L.X.C.); cameron.sknr@outlook.com (C.S.); amanda.edgar@deakin.edu.au (A.K.E.); 2 Deakin Learning Futures, Deakin University, 1 Gheringhap Street, Geelong 3220, Australia

**Keywords:** anterior chamber depth, smartphone, optometry, AS-OCT, Smith’s technique, angle closure glaucoma

## Abstract

**Background:** Measurement of anterior chamber depth (ACD) is an important screening method for angle-closure glaucoma risk. We aimed to determine the precision and agreement of a smartphone-based method for measuring ACD when compared with anterior segment optical coherence tomography (AS-OCT) and Smith’s technique. **Methods:** A total of 43 participants (22 female, 21 male) were recruited. The mean age ± standard deviation of participants was 21.9 ± 2.1 years (range = 19–27 years). Three measurements were taken on each participant’s right eye in a random order for each technique. For this comparative study, differences in ACD, test–retest variability of a given method, and Bland–Altman level of agreement were computed to compare differences in performance between all three methods. **Results:** Smith’s technique over-estimated ACD, while smartphone photography under-estimated ACD, when compared to the reference AS-OCT. There was proportional bias between all three techniques. Smith’s technique had the largest degree of variability, whereas there was no statistically significant difference in test–retest variability between AS-OCT and smartphone photography. **Conclusions:** Zamir’s smartphone photography technique could be used for measuring ACD. Although there are some limitations compared to other established approaches, smartphone photography may have potential as a low-cost screening tool for estimating ACD in settings where specialised equipment is not available. Further studies are required to establish diagnostic performance for angle-closure screening in clinically relevant populations.

## 1. Introduction

In many parts of the world, there is limited access to ophthalmic services, which impacts the burden of eye disease [1]. In many healthcare systems, patients may initially present to general practitioners (GPs) or other non-ophthalmic-related health providers before referral to optometrists or ophthalmologists. Patients with primary angle closure or angle-closure glaucoma often present to these settings, yet such practitioners may not be equipped with the specialised ophthalmic instruments required to make an accurate diagnosis, for example, by measuring the anterior chamber depth (ACD). Consequently, they often rely on diagnostic decision-making algorithms to interpret patients’ symptoms, which can lack sufficient reliability for accurate detection and risk significant visual consequences for patients [2].

There are well-established methods in clinical practice to measure ACD for assessing the risk of sight-threatening conditions [3,4,5]. ACD is generally defined as the distance between the corneal endothelium and the anterior lens capsule [6]. This measurement is known to vary across different populations, reducing with age as the crystalline lens increases in size. A slit lamp can be used to objectively estimate the anterior chamber angle and chamber depth using Van Herick’s method or Smith’s technique, respectively [7,8]. In optometric practice, having the ability to measure ACD is important for screening patients who may be at risk of angle closure when they are required to have their pupils dilated [9]. In ophthalmology practice, accurate ACD measurements are required when calculating the required intraocular lens power before cataract surgery, when considering other treatments such as implantable contact lenses, and when assessing the risk of corneal endothelial injury during surgery [10,11].

Advances in ocular imaging and technology have allowed more accurate measurements to be obtained and improved diagnoses for many conditions [12]. For examining ACD, A-scan ultrasound biometry, anterior segment optical coherence tomography (AS-OCT), interferometry such as the Zeiss IOL Master, and Scheimpflug anterior chamber topography have all been used. A-scan ultrasound biometry is an established method, although it has been suggested that the applanation required for this technique may result in shorter anterior chamber depth measurements [13]. AS-OCT has been shown to provide accurate, repeatable and reproducible measurements of ACD and was used as a reference method against which the smartphone and Smith’s technique were compared in this study [14]. The Van Herick technique and Smith’s technique are useful screening measures, but both require access to a slit lamp, which is generally only available in ophthalmic settings [15,16].

Primary care practitioners such as general practitioners (GPs) or those working in regional outreach clinics who do not have access to ophthalmic instruments would benefit from having the ability to screen patients for angle-closure risk. This would be in locations where physicians often do not have access to a slit lamp or OCT and may be reliant on less accurate techniques such as the shadow test, where a penlight illuminates the eye and the ACD is graded based on the resulting shadow [17]. However, the shadow test technique is difficult to quantify and has been shown to have low sensitivity and specificity for angle closure [18].

Improving access to objective measurements of ACD in primary care has the potential to increase diagnostic accuracy and timely referral and treatment, and, as a result, decrease morbidity rates and serious associated ocular conditions [2]. However, significant barriers limit the ability of primary care practitioners to assess these tools, including the availability and portability of equipment, as well as cost. This may be exacerbated in regions with limited access to eyecare services. There is therefore a need for a readily available, cost-effective and easy-to-use method for assessing ACD that can be utilised outside of specialised eyecare settings.

An alternative method developed by Zamir and colleagues suggested that using a smartphone to capture images could produce repeatable and reproducible measurements of ACD [19]. This has the potential to be a more accurate technique than the shadow test when working in situations where a slit lamp or other specialty ophthalmic instrumentation is not available, as the shadow test can only provide an estimate of ACD and is more subjective than the smartphone method.

To assess the accuracy of this smartphone-based method compared to more established conventional methods, this study aimed to compare ACD measured by Zamir’s smartphone photography technique to AS-OCT and Smith’s technique. Smith’s technique was selected for comparison in the present study because it provides a quantitative estimate of central anterior chamber depth in millimetres, allowing for direct comparison with AS-OCT and smartphone ACD measurements. In contrast, the Van Herick technique estimates peripheral anterior chamber angle width rather than central ACD and therefore was not directly comparable to the primary outcome measured in this study. This is the first study of its kind to compare Smith’s technique, AS-OCT and smartphone photography together. The primary aim of our study was to compare agreement and repeatability between measurement techniques rather than to evaluate angle-closure screening.

## 2. Materials and Methods

### 2.1. Ethics

Ethical approval was granted by the Deakin University Human Research Ethics Committee (HEAG-H 114_2018). The study was set in the optometry teaching clinic at Deakin University, Waurn Ponds, Australia, and conducted in accordance with the tenets of the Declaration of Helsinki. Before enrolment, participants were given information regarding the purpose of the study, and written informed consent was obtained prior to study enrolment.

### 2.2. Population

A total of 43 participants (22 female, 21 male) were recruited for the study. The mean age ± standard deviation of participants was 21.9 ± 2.1 years (range = 19 to 27 years). Participants were recruited from a university population as a convenience sample for this comparison study. The mean refractive error equivalent sphere for the group was −1.04 DS ± 2.38 DS (range = −10.50 DS to + 2.00 DS). For inclusion in the study, participants were required to be aged 18 years or older and have no systemic or ocular disease that may confound the study results. Participants were excluded if they had an ocular history of cataract, corneal abnormalities or ectasias such as keratoconus, any previous penetrating eye injury that would affect the anterior chamber, or temporal limbal eye lesions, or if they were using orthokeratology contact lenses.

### 2.3. Measurement Method

In this study, the ACD was defined as the distance between the corneal endothelium and the anterior capsule of the lens at the corneal apex (the central point of the cornea) [20]. All participants underwent three randomised procedures sequentially on the same visit: 1. AS-OCT; 2. Smith’s technique; 3. smartphone photography. Three measurements were obtained of each participant’s right eye per technique, totalling nine measurements per participant. The order of measurement technique for each participant was randomised. All researchers performing measurements were optometry students who had completed training in the relevant clinical assessment procedures.

Two researchers were allocated to each measurement technique and were masked to the results of the other techniques. Prior to the start of the main study, a pilot study was performed involving two participants and six researchers to assess the repeatability and agreement between assessors across the three measurement techniques. Researchers with the most repeatable results and highest inter-observer agreement were allocated to a measurement technique. Formal inter-observer agreement analysis was not the primary outcome of the present study, as Bland–Altman analysis was used to assess agreement between measurement techniques rather than between assessors.

### 2.4. AS-OCT

**Figure 1:**
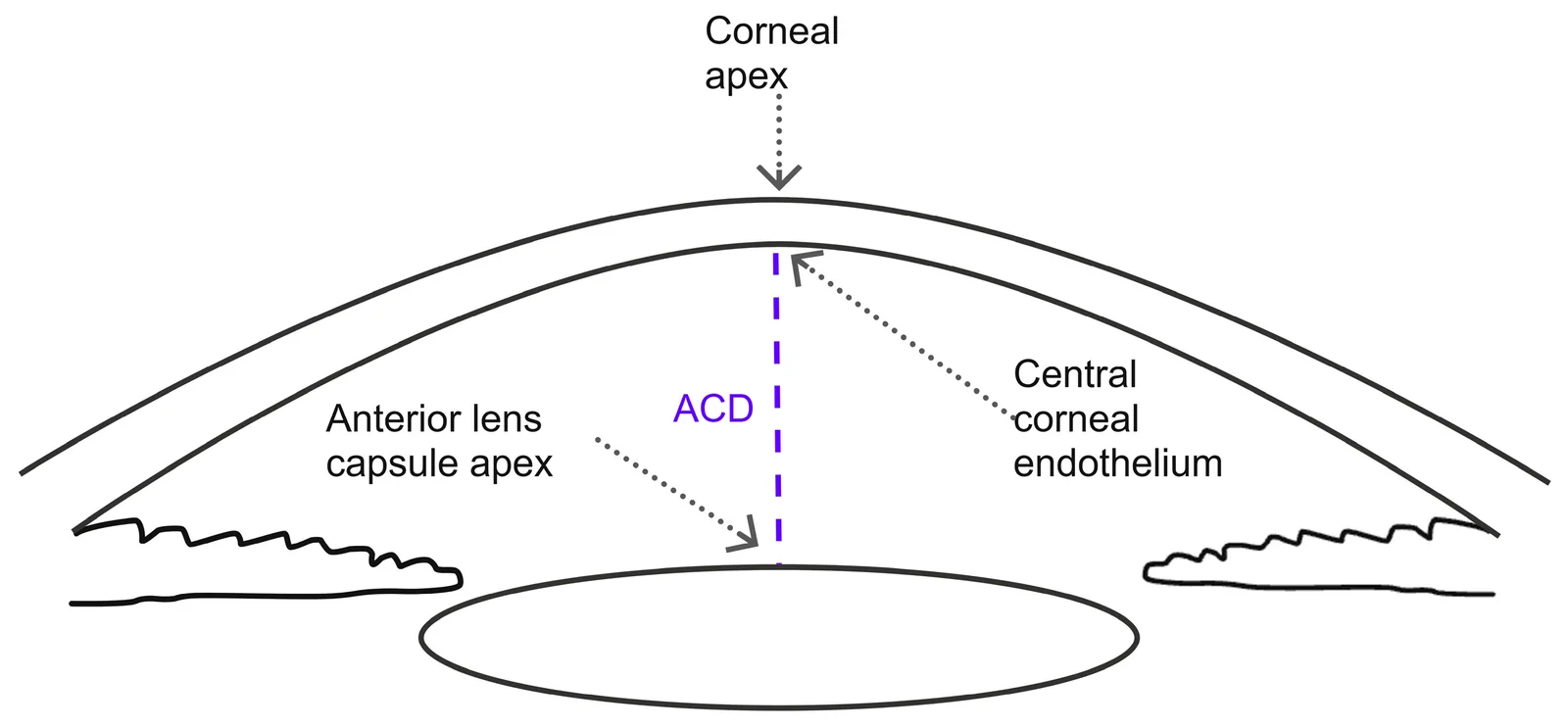
Schematic illustration of anterior chamber depth measurement using anterior segment optical coherence tomography. ACD (blue dashed line) was measured using built-in callipers as the distance between the central corneal endothelium and the anterior lens capsule at the corneal apex.

The Zeiss CIRRUS AS-OCT (Carl Zeiss Meditec AG, Jena, Germany) was used to obtain three images of the anterior segment along the horizontal midline of each participant’s right eye. The ACD was measured directly from the image using the built-in callipers provided by Zeiss CIRRUS review software version 11.5.2.54532 (Figure 1). The central point of the cornea was identified on the screen image, and the measurement callipers were manually placed with one point at the central point of the corneal endothelium and the second at the apex of the anterior capsule of the crystalline lens. One reading was taken from each of the three images, and the average of these three measurements was used for calculations.

### 2.5. Smith’s Technique

**Figure 2:**
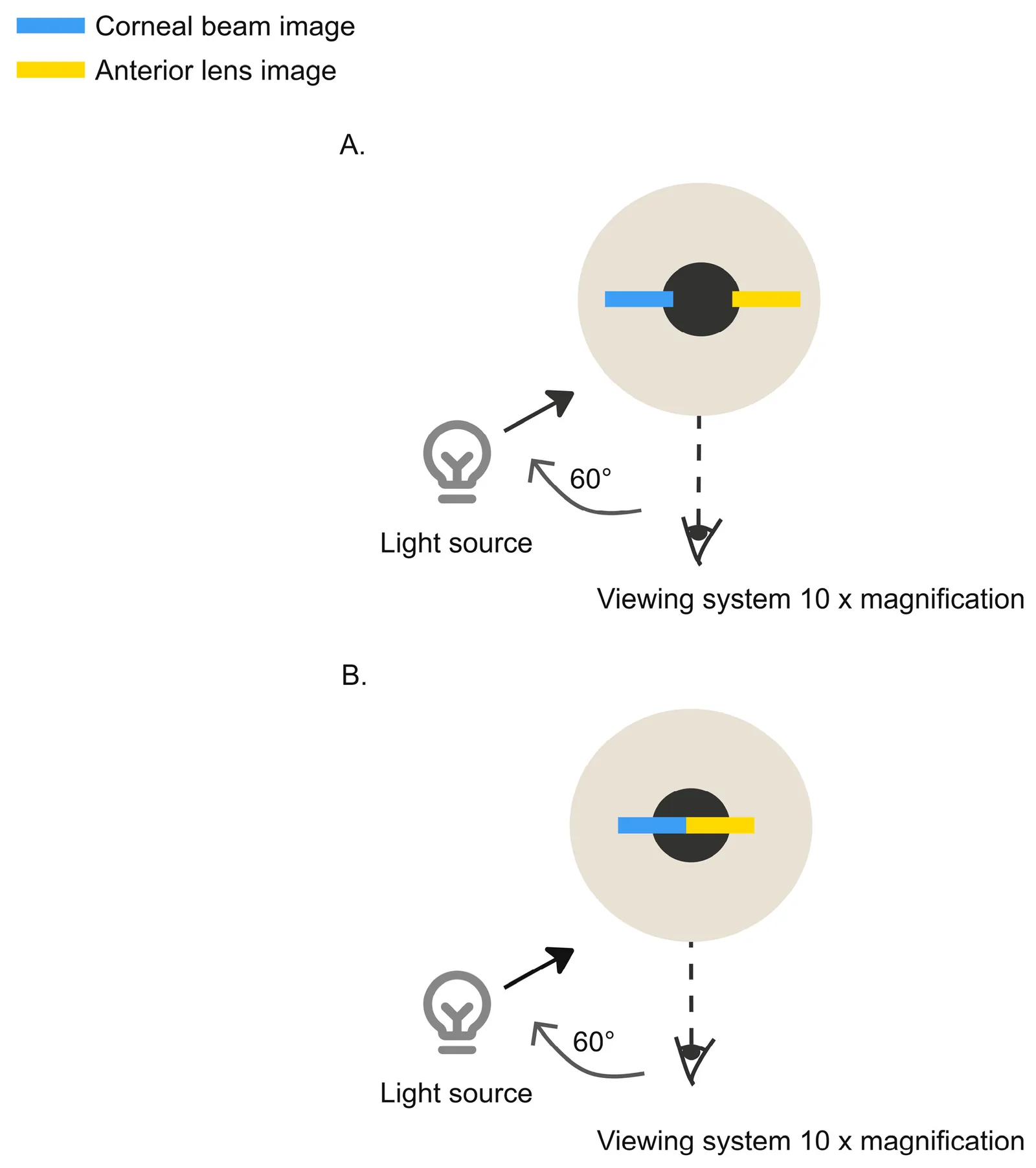
Schematic illustration of Smith’s technique for estimating anterior chamber depth (ACD). (**A**) Corneal and anterior lens beam images are separated. (**B**) Corneal and anterior lens beam images just touch, representing the measurement endpoint. The slit-lamp biomicroscope observation system was positioned directly in front of the eye at 10× magnification, while the illumination system was positioned 60° temporally. A horizontal slit beam (1.5 mm width) was focused on the central cornea.

For Smith’s technique, a Haag-Streit slit lamp (Haag Streit, Köniz, Switzerland) was used to view the anterior eye and the technique as described by RJ Smith was used to estimate the anterior chamber depth using a conversion factor of 1.34 [8,21] (Figure 2).

The biomicroscope was positioned directly in front of the patient’s eye with the illumination system angled 60 degrees temporally from the observation system. Magnification was set to 10×, and participants were instructed to fixate on an illuminated green cross 3 m away to minimise accommodation. A 1.5 mm horizontal slit beam was focused on the central cornea. A second horizontal beam image was observed in the plane of the crystalline lens (Figure 2A), and the beam length was adjusted until the corneal and anterior lens beam images just touched (Figure 2B). Three measurements of beam length were obtained, and ACD was calculated using Equation (1) [8] below:

ACD = (slit lamp beam length) × 1.34(1)

### 2.6. Smartphone Photography

Smartphone photography of the anterior chamber was carried out in accordance with the methods described by Zamir and colleagues [19]. An accommodative target consisting of an illuminated green cross was placed at 3 m from participants, who were instructed to focus on this target during the procedure.

An iPhone 6s (iOS version 11.2.2, Apple Inc. Los Altos, CA, USA; 12-megapixel rear camera, f/2.2 aperture, 29 mm equivalent focal length) was placed on a tripod 10 cm away from the participant’s lateral canthus using a set-square to ensure the height of the phone was aligned with the lateral canthus and the participant’s visual axis. A spirit level was then used to ensure that the phone was level, and the researcher checked that the corneal and limbal structures were in focus. The phone was then rotated 90 degrees to the limbus using a square ruler. The flash setting was turned on, and participants were instructed to open their eyes as wide as possible and to occlude the eye that was not being photographed while continuing to fixate on the target.

**Figure 3:**
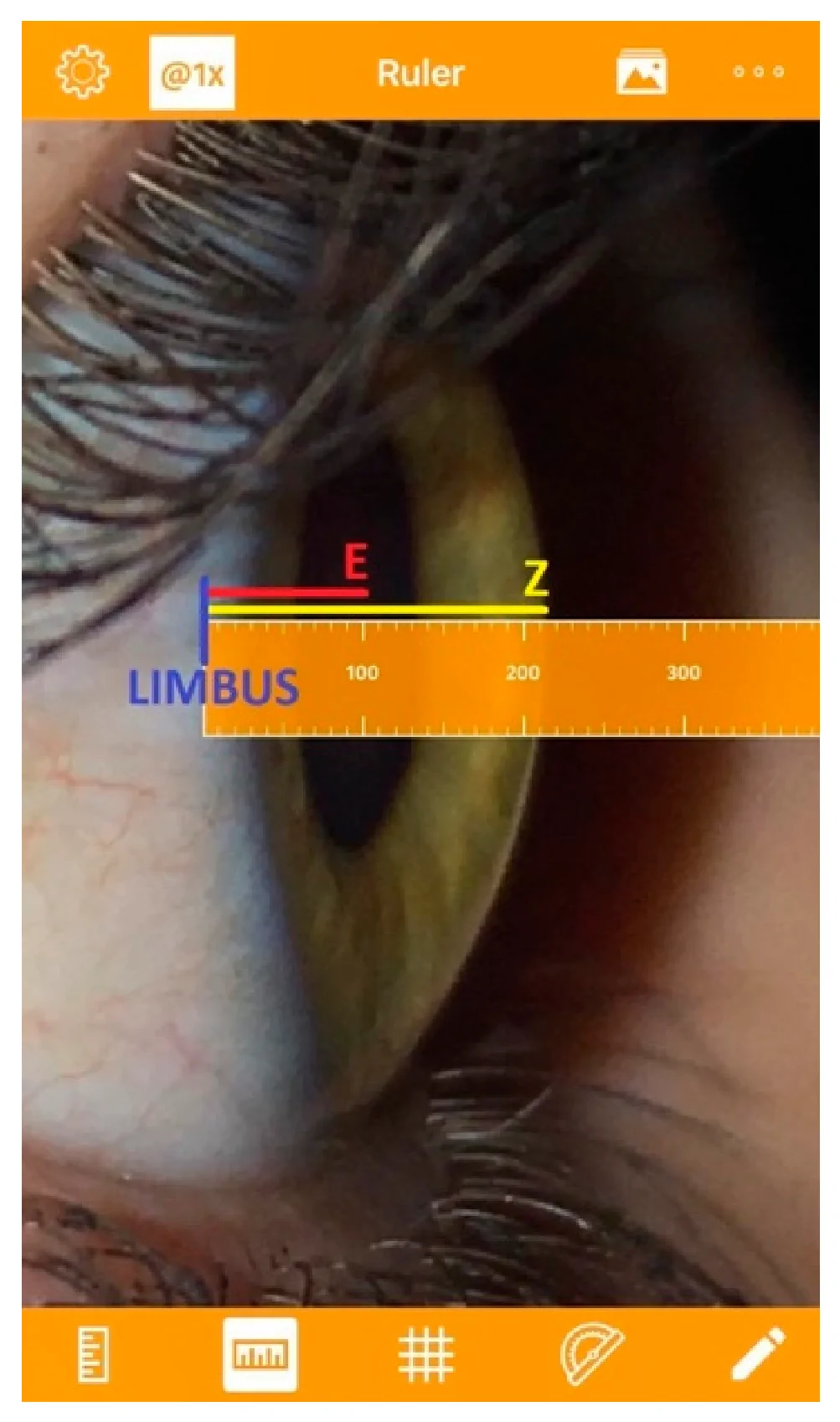
Image with measurements E and Z using smartphone with pixel ruler application.

The image was then taken using a Bluetooth camera shutter to minimise any camera shake. After the image was taken, the quality was immediately assessed by the researcher, and if inadequate for analysis, a second photograph was taken. Three individual photographs of acceptable quality were obtained for each participant. Photographic images were analysed using the Pixel Ruler application on the iPhone (APKpure International Limited, Singapore, Singapore). Measurements were taken of the distance from the limbus to the middle of the pupil (E) and the distance from the limbus to the apex of the cornea (Z) for each of the three photos (Figure 3).

The E:Z ratio (EZR) was derived from these two measurements, and the anterior chamber depth was calculated using Equation (2) [19]. For example, if the EZR is 0.5, then ACD = (−3.273 × 0.5) + 4.18 = 2.55 mm.

(2) ACD=−3.273(EZR)+4.18

### 2.7. Statistical Analysis

The data were analysed using SPSS statistical software (Version 29, IBM, New York, NY, USA). Descriptive statistics were used to determine the mean and range. A repeated-measures ANOVA was performed to investigate differences in mean anterior chamber depth between AS-OCT, smartphone photography and Smith’s technique. Bland–Altman plots were used to assess the level of agreement between techniques [22,23,24]. In addition, 95% limits of agreement (LoAs) were computed (±1.96 × standard deviation of the differences in measurement). Proportional bias was calculated using a linear regression on the Bland–Altman plots.

To investigate test–retest variability, the test–retest standard deviation (TRT-SD) was calculated across the three repeated measurements for each measurement technique and for each participant. Mean TRT-SD values were compared across techniques using one-way repeated-measures ANOVA, with Bonferroni-corrected post hoc paired t-tests to identify any significant differences between measurement techniques [24]. In this study, statistical significance was determined as *p* < 0.05.

## 3. Results

### 3.1. Comparison of ACD Measurements

**Figure 4:**
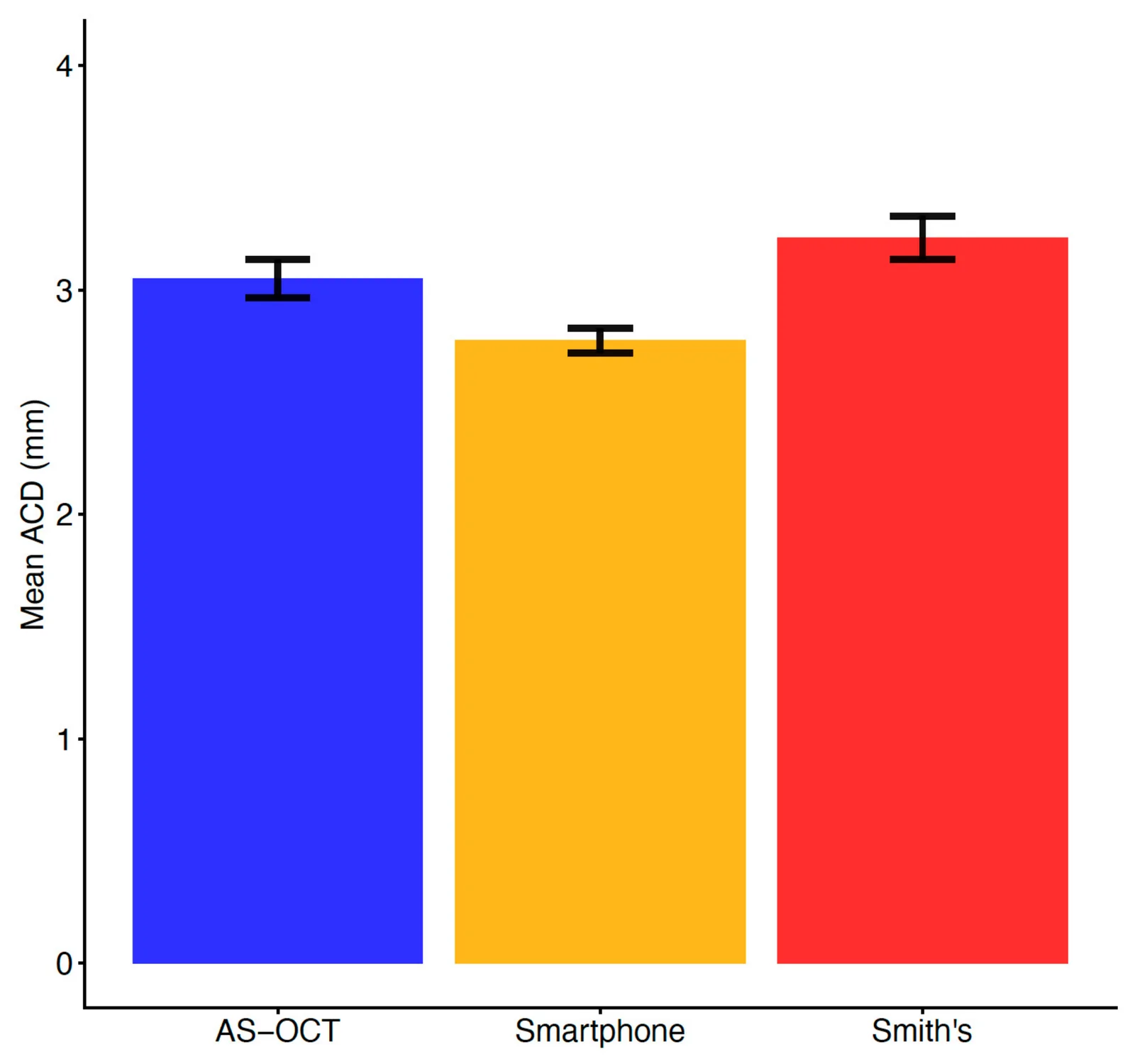
Mean anterior chamber depth of AS-OCT, smartphone photography and Smith’s technique. Error bars represent the 95% confidence interval.

The mean ACD measured by AS-OCT was 3.05 ± 0.29 mm. Smith’s technique resulted in larger ACD measurements compared to AS-OCT, with a mean of 3.23 ± 0.32 mm, while the smartphone photography technique gave a smaller mean measurement of 2.78 ± 0.32 mm (Figure 4). Differences observed in mean ACD between all three measurement techniques were statistically significant (*p* < 0.001, repeated-measures ANOVA), with Bonferroni-corrected post hoc pairwise comparisons showing significant differences between all techniques (all *p* < 0.001).

**Figure 5:**
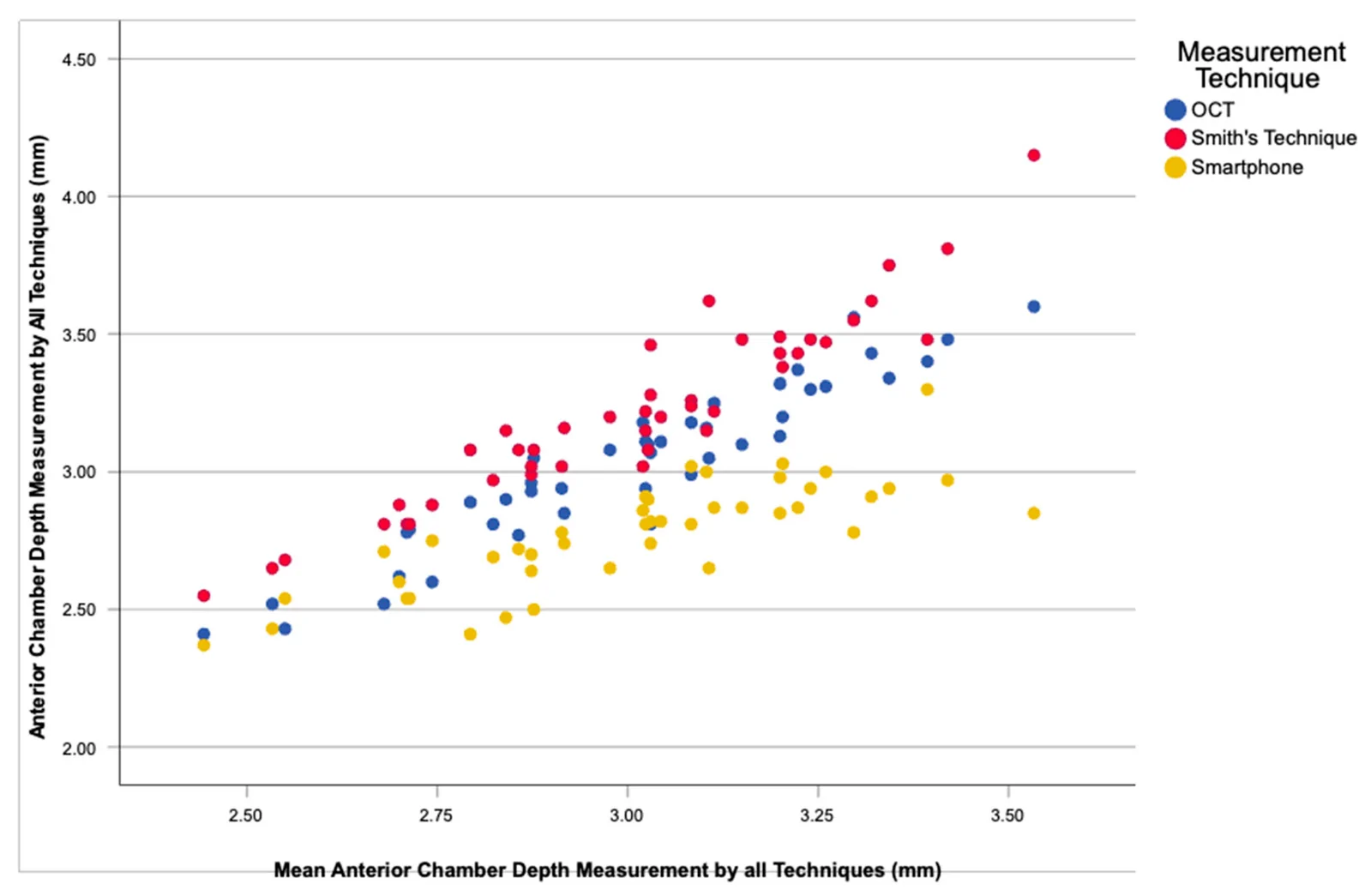
ACD measurements of AS-OCT, Smith’s technique and smartphone photography stratified by average ACD across all techniques.

We observed the same trend when comparing ACD measurements stratified by mean ACD, with Smith’s technique resulting in larger ACD measurements and smartphone photography resulting in smaller ACD measurements relative to AS-OCT as the reference (Figure 5). It also showed a closer agreement between measurements for shallower anterior chambers and a greater spread for deeper anterior chambers. However, it can also be observed that smartphone photography demonstrated more proportional bias with a greater difference compared to both AS-OCT and Smith’s technique in participants with deeper ACD measurements.

### 3.2. Test–Retest Variability

**Figure 6:**
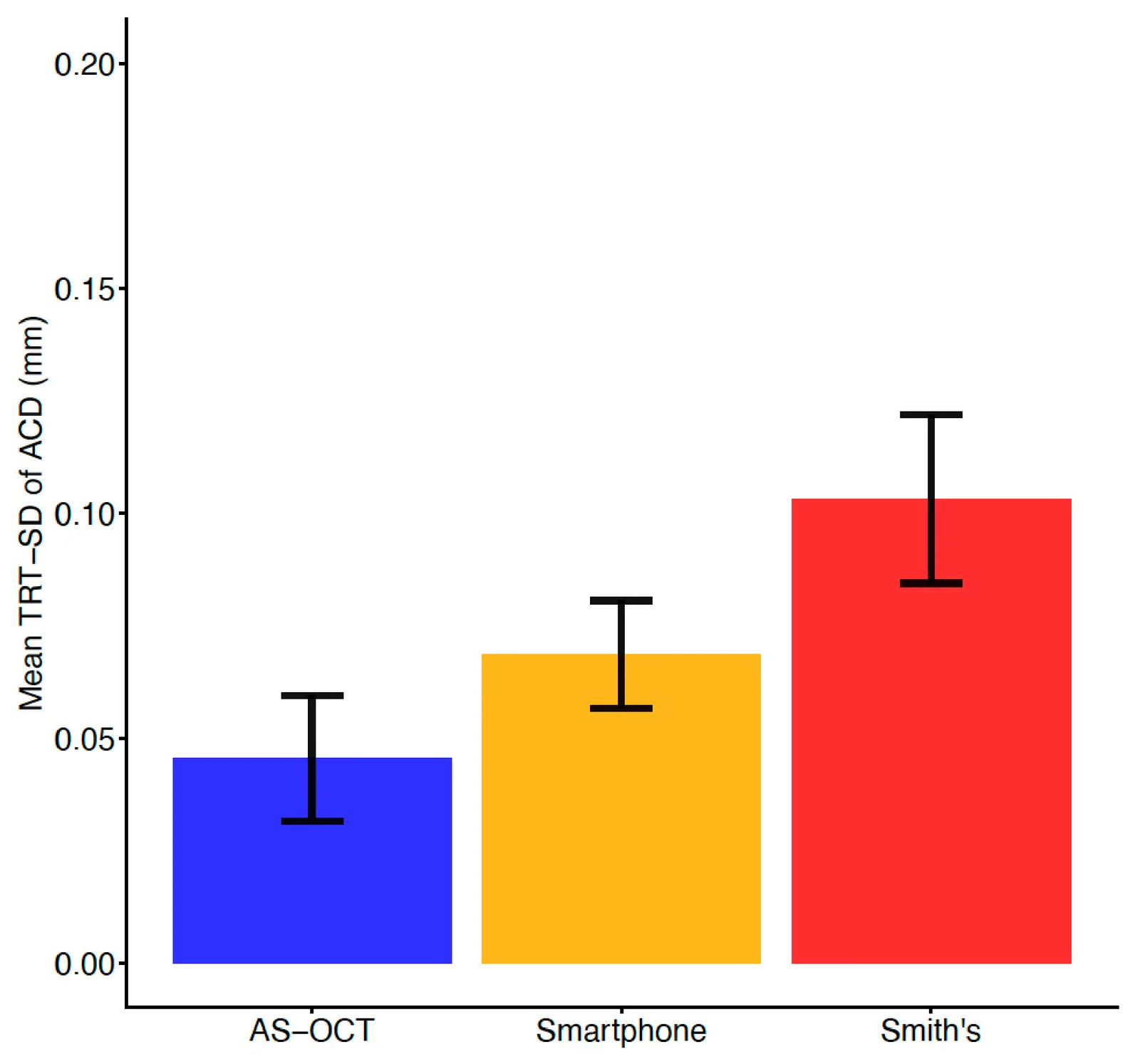
Mean test–retest standard deviation of anterior chamber depth of AS-OCT, smartphone photography and Smith’s technique. Error bars represent the 95% confidence interval.

The mean ± SD of TRT-SD was 0.045 ± 0.047 for AS-OCT, 0.069 ± 0.040 for smartphone photography and 0.103 ± 0.063 for Smith’s technique (Figure 6). The repeated-measures ANOVA revealed that there was a significant difference in mean TRT-SD between at least one pair of measurement techniques (*p* < 0.001). Post hoc pairwise t-tests with Bonferroni correction revealed that there was a significant difference between AS-OCT and Smith’s technique (*p* < 0.001), and between smartphone photography and Smith’s technique (*p* = 0.012). Although AS-OCT demonstrated lower variability than smartphone photography, the difference did not reach statistical significance after Bonferroni correction (*p* = 0.071).

### 3.3. Bland–Altman Level of Agreement

**Figure 7:**
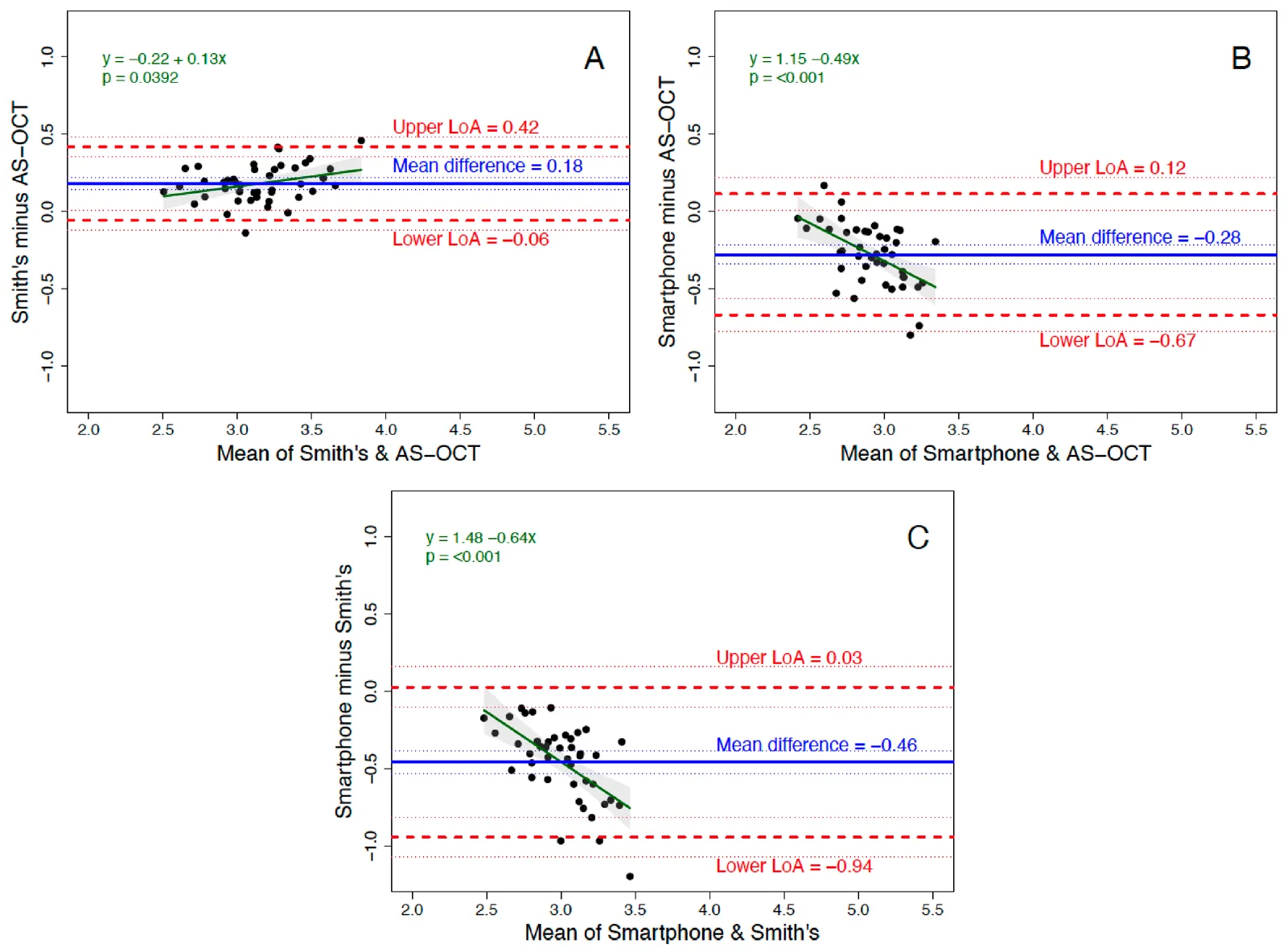
Bland–Altman plots showing the mean difference and limits of agreement (LoAs) with their 95% confidence intervals of ACD measurements between (**A**) Smith’s technique and AS-OCT, (**B**) smartphone photography and AS-OCT, and (**C**) smartphone photography and Smith’s technique. Plots include proportional bias with 95% confidence intervals for each technique comparison (indicated with grey shading), along with the linear regression line of best fit (solid green lines), regression formulae and associated *p*-value (green text).

The level of agreement between the three techniques is illustrated by the Bland–Altman plots in Figure 7. Smith’s technique demonstrated the narrowest level of agreement with AS-OCT, with a mean difference of 0.18 mm and width of the 95% limits of agreement (LoAs) of 0.48 mm. Smartphone photography and AS-OCT had the second highest agreement, with a mean difference of 0.28 mm and 95% LoA width of 0.79 mm. There was relatively weak agreement between Smith’s technique and smartphone photography, with a mean difference of 0.46 mm and 95% LoA width of 0.97 mm.

Proportional bias was plotted using a linear regression on the Bland–Altman plots. There was significant proportional bias between all three techniques (Smith’s vs. AS-OCT, β = −0.316, *p* = 0.039; smartphone vs. AS-OCT, β= 0.532, *p* < 0.001; smartphone vs. Smith’s, β = −0.605, *p* < 0.001). Smartphone photography underestimated ACD for deeper anterior chambers when compared to AS-OCT; however, this difference was smaller for shallower anterior chambers.

## 4. Discussion

This study found that ACD measurements taken using the smartphone photography technique demonstrated acceptable repeatability and moderate agreement with AS-OCT and Smith’s technique in our study sample. Although measurements were statistically different between techniques, statistical significance does not necessarily translate to clinical significance; the clinical implications of these differences remain uncertain and require investigation in clinically relevant populations. Average ACD measurements for AS-OCT and Smith’s technique were comparable to previously published data [25]. The mean ACD by AS-OCT in this study measured 3.05 ± 0.29 mm while Dominguez-Vicent found an average ACD measurement of 3.08 mm with AS-OCT in a slightly older European population with a similar refractive error (−1.19 DS) [25]. Although Yi and associates found an average measurement of 3.32 mm using AS-OCT in an Asian population of similar age, the larger ACD in their study may potentially be due to their more myopic population, which was −3.70 DS compared to −1.03 DS in our study [26]. Measurements using Smith’s technique were also comparable with an average ACD of 3.22 mm reported by Eperjesi and Holden in a similar population. Overall in this study, Smith’s technique gave deeper ACD measurements compared to AS-OCT. This may be due to the approximations used in the measurement technique, with the slit-lamp beam measurement only able to be measured to within 1.0 mm using the measurement scale on the slit lamp. Smartphone photography, on the other hand, gave shallower AC depth measurements when compared with the other two techniques. However, ACD measurements from the smartphone technique were of a similar magnitude to those reported by Zamir and colleagues, suggesting reasonable consistency of the technique across studies [19].

Although the smartphone method gave a measurement that was statistically different to AS-OCT, readings obtained with this method were consistently smaller, suggesting that the equation used may need to be recalibrated. Zamir and colleagues used a Canon digital camera to take their images, while for this study we used an iPhone. This may have resulted in a slight difference in pixels affecting the measurement. Subsequent studies will be needed to investigate different phone camera pixels and compare readings taken from each. However, despite this limitation, the smartphone technique demonstrated reasonable repeatability and moderate agreement with AS-OCT and Smith’s technique and may warrant further investigation as a low-cost method for estimating ACD where specialised ophthalmic equipment is unavailable. Smartphone measurements demonstrated reasonable repeatability but lower agreement with AS-OCT than Smith’s technique.

There was modest overall agreement between the three techniques, as shown by the Bland–Altman plots in Figure 7. Although Smith’s technique showed the narrowest limits of agreement with AS-OCT, significant proportional bias was also observed, including that agreement varied across the range of ACD values. There was also moderate agreement between AS-OCT and the smartphone method. The lowest agreement was between Smith’s technique and smartphone photography. This was likely due to the requirement to calculate these measurements as opposed to direct measurement of the ACD with AS-OCT, potentially increasing any measurement error. Having a quantifiable method of assessing angle-closure risk allows for follow-up over time more readily than using a non-quantifiable screening measurement such as the shadow test, where the exact ACD cannot be measured. Screening tests such as Smith’s technique have been used successfully to determine risk of angle closure [16], and where a slit lamp is not available, the smartphone technique could provide a viable alternative.

When compared to Smith’s technique and AS-OCT, there was significant proportional bias when using the smartphone method, with the difference from AS-OCT increasing across the range of ACD values. This finding suggests that the measurement error of the smartphone technique varies according to ACD rather than remaining constant across a measurement range. Therefore, while agreement is stronger for shallow ACD, the technique underestimated ACD as chamber depth increased. Similar bias was observed when smartphone measurements were compared with Smith’s technique. These findings indicate that the smartphone method should not currently be considered interchangeable with AS-OCT measurements. Although this bias was significant, agreement was greater for the shallower ACD measurements. However, as clinicians are generally more concerned about shallow ACDs rather than deep ACDs when screening for angle-closure risk, this limitation of underestimating deeper ACDs may not be as relevant in clinical settings. However, there is a risk of an increase in false-positive rate with the smartphone technique as a result of this bias. It should also be noted that significant proportional bias was observed between Smith’s technique and AS-OCT; however, the magnitude of disagreement and limits of agreement were smaller than those observed for the smartphone method.

The smartphone method of measuring ACD was found to be reliable with a TRT-SD of 0.069 ± 0.040. This was comparable to the AS-OCT test–retest variability of 0.045 ± 0.047 and was more precise than Smith’s technique, which had a TRT-SD of 0.103 ± 0.063. Smith’s technique requires more operator input and precise determination of endpoints to obtain the measurement, which may contribute to the higher variability found with these measurements. Smartphone photography requires less clinical expertise to operate, and provided a photograph is obtained with adequate quality and resolution, measurements can be taken with measurement software applications such as Pixel Ruler, thereby reducing human error.

In practice, ACD measurement is used as a screening method for the risk of angle closure and angle-closure glaucoma [27]. Since angle-closure suspects have narrow angles and a shallow ACD, the greater agreement between AS-OCT and the smartphone method for calculating ACD in shallower angles may warrant further investigation to ensure that such patients are identified and not missed using the smartphone method [28,29]. The bias towards a shallower anterior depth measurement compared to AS-OCT and Smith’s technique may result in patients being referred for further examination who are not at risk of angle closure, but is less likely to miss any patients with a true shallow angle. A greater accuracy in ascertaining shallow ACD may therefore potentially be preferred as a screening method for risk of angle closure.

Measurements for all techniques were found to be repeatable. AS-OCT showed the highest degree of repeatability followed by the smartphone photography technique. Smith’s technique demonstrated the most variability out of the three techniques that were compared.

There were some limitations in this study other than those previously mentioned. Firstly, the study assesses measurement performance in healthy young adults rather than in individuals at risk of angle closure. The younger population had deeper anterior chambers, and the lower sample size of shallower ACD may have influenced the results. Future studies with a larger sample size and a more diverse range of ACDs, such as including a study sample of those with shallow ACDs (<2 mm) or a history of primary angle-closure glaucoma, would help determine if the findings obtained in this study would also apply to participants with narrower ACDs. However, these findings should be interpreted cautiously given the limited representation of participants with shallow anterior chamber angles. There is a further need to validate these findings within clinically relevant populations and clinical settings before conclusions can be made in regard to screening practice and performance. Importantly, the present study evaluated measurement agreement and repeatability rather than diagnostic utility. Further studies should investigate sensitivity, specificity and overall diagnostic performance of the smartphone technique against clinically relevant reference standards, including gonioscopy, in populations at risk of angle closure. Additionally, more modern smartphones with newer cameras may require calibration, as additional lenses, the use of multiple images and artificial intelligence to produce image-corrected photographs and newer optical zooms may impact the original equation proposed by Zamir et al. [30]. In addition, variations in eye-to-camera distances and image acquisition procedures may affect the EZR measurement and therefore estimated ACD.

## 5. Conclusions

In conclusion, the smartphone photography technique demonstrated good repeatability and consistently produced shallower ACD estimates than AS-OCT and Smith’s technique. As agreement with AS-OCT was lower than that observed with Smith’s technique and significant proportional bias was present at deeper ACD measurements, the results from this study suggest that traditional methods of measuring ACD such as AS-OCT and Smith’s technique perform better than the smartphone photography method, provided that the required equipment is available and the operator is appropriately trained. However, the smartphone photography method showed potential as a low-cost screening approach for estimating ACD where specialised ophthalmic equipment is unavailable. The present findings support the feasibility and repeatability of smartphone ACD measurements in healthy young adults; however, further studies are required to establish diagnostic performance for angle-closure screening in clinically relevant populations. Recalibration of the original Zamir equation may also provide agreement with contemporary imaging devices.

## Data Availability

The dataset generated and analysed during the current study is available to the authors but is not publicly available due to ethical guidelines. The datasets used and/or analysed during the current study are available from the corresponding author on reasonable request.

## Abbreviations

The following abbreviations are used in this manuscript:

ACD	Anterior chamber depth
AS-OCT	Anterior segment optical coherence tomography
SD	Standard deviation
TRT-SD	Test–retest standard deviation
GPs	General practitioners
EZR	E:Z ratio
LoA	Limit of agreement
